# Mechanistic insights into the regression of atherosclerotic plaques

**DOI:** 10.3389/fphys.2024.1473709

**Published:** 2024-11-19

**Authors:** Jianshu Song, Ce Cao, Ziyan Wang, Haoran Li, Lili Yang, Jing Kang, Hongxu Meng, Lei Li, Jianxun Liu

**Affiliations:** ^1^ National Research Center for Clinical Medicine of Cardiovascular Diseases of Traditional Chinese Medicine, Beijing Key Laboratory of Traditional Chinese Medicine Pharmacology, Xiyuan Hospital, Chinese Academy of Traditional Chinese Medicine, Beijing, China; ^2^ Research Institute of Traditional Chinese Medicine of Guangdong Pharmaceutical University, Guangzhou, China

**Keywords:** atherosclerosis, plaque regression, reverse cholesterol transport, monocyte/macrophage, macrophage polarization, macrophage efferocytosis

## Abstract

Atherosclerosis is a major contributor to cardiovascular diseases and mortality globally. The progression of atherosclerotic disease results in the expansion of plaques and the development of necrotic cores. Subsequent plaque rupture can lead to thrombosis, occluding blood vessels, and end-organ ischemia with consequential ischemic injury. Atherosclerotic plaques are formed by the accumulation of lipid particles overloaded in the subendothelial layer of blood vessels. Abnormally elevated blood lipid levels and impaired endothelial function are the initial factors leading to atherosclerosis. The atherosclerosis research has never been interrupted, and the previous view was that the pathogenesis of atherosclerosis is an irreversible and chronic process. However, recent studies have found that the progression of atherosclerosis can be halted when patients’ blood lipid levels are reversed to normal or lower. A large number of studies indicates that it can inhibit the progression of atherosclerosis lesions and promote the regression of atherosclerotic plaques and necrotic cores by lowering blood lipid levels, improving the repair ability of vascular endothelial cells, promoting the reverse cholesterol transport in plaque foam cells and enhancing the ability of macrophages to phagocytize and clear the necrotic core of plaque. This article reviews the progress of research on the mechanism of atherosclerotic plaque regression. Our goal is to provide guidance for developing better therapeutic approaches to atherosclerosis by reviewing and analyzing the latest scientific findings.

## 1 Introduction

Cardiovascular disease, especially ischemic heart disease, remains the leading cause of mortality and morbidity (Saglietto et al., 2021; Vaduganathan et al., 2022; Joseph and Yusuf, 2023). Atherosclerosis (AS) is the main pathological process of most cardiovascular diseases (Madaudo et al., 2024). AS is a chronic pathological change in the arterial wall layer, affecting the vascular system throughout the body. AS can cause local inflammatory reactions, vascular stenosis, and even blockage of blood vessels by thrombus plaques, leading to insufficient blood supply to end-organ tissues, and thus inducing ischemic damage (Altabas and Biloš, 2022). The mechanism of AS is an extremely complex physiological and pathological process, which has not yet been fully elucidated. The current mainstream view is that vascular endothelial damage, dysfunction, and inflammatory response are the initiating factors of AS (Mudau et al., 2012; Ruan et al., 2013; Souilhol et al., 2018). The permeability of the vascular endothelium increases leading to lipids in the blood, especially low-density lipoprotein (LDL), flowing into and depositing in the intima when the vascular endothelium is damaged. Then they are oxidized into oxidized low-density lipoprotein (ox-LDL), causing an inflammatory response. In this context, monocytes are continuously recruited to the vascular endothelium, and infiltrating monocyte-derived macrophages, as well as lesional macrophages internalize ox-LDL to generate foam cells, and form lipid streaks in the early stage of AS lesions (Yu et al., 2013). Foam cells, together with vascular smooth muscle cells (VSMCs), produce a variety of pro-inflammatory mediators, including matrix metalloproteinases (MMPs), interleukin-6 (IL-6), and C-reactive protein (CRP), which further amplify the inflammatory response, trigger secondary cell necrosis, and then develop into a necrotic core (Falk, 2006; Wolf and Ley, 2019; Attiq et al., 2024). Thrombotic vascular blockage caused by plaque rupture in the late stage of AS can easily lead to complications such as stroke and myocardial infarction, which brings great pain and harm to patients (Blagov et al., 2023).

Past studies have generally believed that the pathogenesis of AS is an irreversible chronic process (Williams et al., 2008). However, studies in recent years have shown that reducing plasma lipoprotein and cholesterol concentrations to normal levels improves AS symptoms to some extent and may even lead to the regression of plaques (Mendieta et al., 2023). More and more studies and evidence indicate that the development of AS can be inhibited to increase the stability and promote regression of AS plaque by reducing cholesterol concentration, anti-inflammation, and other pathways (Kaul et al., 2004; Dawson et al., 2022), which suggests a promising future for anti-atherosclerotic therapy. It is important to note that plaque regression is not simply a reversal of plaque progression. The regression of plaque means that the arterial wall will return to its original state in an ideal situation. However, it is impossible to completely reverse the development of plaques. Current research suggests that the mechanism of plaque regression mainly involves the following key processes: promoting endothelial repair, increasing the cholesterol efflux capacity from foam cells or macrophages, and clearing or reducing necrotic cell fragments in the necrotic core (Manubolu and Budoff, 2022).

## 2 Pathogenesis of AS

In the early stages of atherosclerotic lesions, the binding of small lipoprotein particles to proteoglycans on the intima surface results in their retention in the intima for a longer time (Little et al., 2021). Lipoprotein particles bound to proteoglycans are more susceptible to oxidation and other chemical modifications, leading to chronic inflammation. Additionally, the increased permeability of endothelial monolayer to LDL further promotes the accumulation of lipoprotein in vascular intima and the inflammatory expression of endothelial cells, as manifested by increased expression of vascular cell adhesion molecule (VCAM-1), intercellular adhesion molecule (ICAM-1), E-selectin and monocyte chemoattractant protein 1 (MCP-1) (Zhu et al., 2018; Dri et al., 2023). It triggers the recruitment of circulating monocytes to the intimal surface and migrate directionally through the vascular endothelium into the arterial wall under the action of MCP-1, phagocytizing lipids through scavenger receptor A1 (SR-A1), SR-B3 (CD36) and lectin-like oxidized low-density lipoprotein receptor-1 (LOX1) to form foam cells (Yu et al., 2013; Chistiakov et al., 2017a). The appearance of foam cells is a cytological feature of early plaque lesions in atherosclerosis.

In addition to endothelial cells and monocytes, vascular smooth muscle cells (VSMCs) also play a crucial role in the progression of AS. In healthy vascular walls, VSMCs exhibit a more contractile phenotype characterized by the high expression of smooth muscle α-actin (ACTA2), smooth muscle myosin heavy chain (MYH11), 22-kDa smooth muscle cell (SMC) lineage-restricted protein (SM22α/tagln), etc., which are regulated by myocardin (MYOCD) (Bennett et al., 2016; Durham et al., 2018; Frismantiene et al., 2018; Grootaert and Bennett, 2021). However, in the context of AS and vascular injury, they transform into a synthetic phenotype mediated by platelet-derived growth factor BB (PDGF-BB) and Krüppel-like factor family member 4 (KLF4). This phenotype is characterized by high expression of extracellular matrix (ECM), matrix metalloproteinases (MMP), and pro-inflammatory factors (Grootaert and Bennett, 2021), along with high expression of myosin isomers of embryonic VSMCs. Instead of myosin specific to mature smooth muscle cells, the cell morphology changes from spindle to rhomboid or flattened (Zhang et al., 2022). During disease progression, a key biological change of SMCs is the transition from a contractile phenotype (differentiated, quiescent, non-migratory) to a synthetic phenotype (dedifferentiated, proliferative, migratory), which significantly contributes to the early diffuse intimal thickening (DIT) and pathological intimal thickening (PIT) of AS, thereby accelerating the progression of AS (Frismantiene et al., 2018; Elmarasi et al., 2024). In addition, synthetic phenotype VSMCs also can differentiate into macrophage-like cells expressing macrophage markers leading to increased uptake of ox-LDL under the vascular intima and subsequent formation and accumulation of foam cells. Furthermore, VSMC-derived macrophage-like cells exhibit reduced phagocytic ability and weakened clearance of lipids and cell debris compared to classical macrophages, resulting in the accumulation of cell debris that promotes local inflammation and the formation of necrotic cores, ultimately contributing to the progression of AS lesions (Allahverdian et al., 2014; Yap et al., 2021; Chen et al., 2023).

**FIGURE 1:**
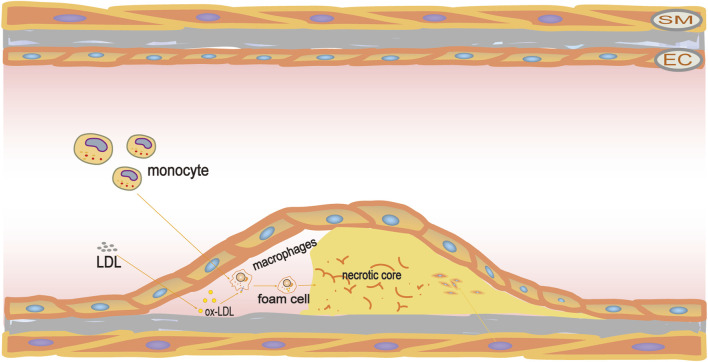
Pathogenesis of atherosclerosis. LDL travels through the vascular endothelium and accumulates beneath the intima, where it undergoes oxidation to become ox-LDL. Following this, macrophages ingest the ox-LDL to transform into foam cells. These foam cells proceed to amass and experience apoptosis and necrosis, resulting in the creation of a necrotic core and ultimately contributing to the development of atherosclerotic plaques.

Eventually, the accumulated foam cells undergo apoptosis, resulting in the formation of a necrotic core. During the early stages of AS development, apoptotic foam cells are typically cleared by phagocytic macrophages through a process known as macrophage efferocytosis (Li et al., 2021). However, excessive ingestion of apoptotic cells by macrophages may eventually put pressure on the endoplasmic reticulum stress. It will lead to dysfunction of macrophage efferocytosis and failure to clear apoptotic cells in time, causing the accumulation of apoptotic cells causing the necrotic core to continue to grow, thus developing into unstable and rupture-prone plaques (Bäck et al., 2019). In addition, the release of substances in cellular contents such as intracellular lipids, pro-inflammatory/pro-thrombotic mediators, and metalloproteinases (MMPs) damages surrounding cells and tissues, leading to plaque rupture, thrombosis, and vascular occlusion events (Badimon and Vilahur, 2014; Baraniecki et al., 2023). (Figure 1)

AS is a multifocal chronic disease process, usually driven by abnormally elevated blood lipid levels (Frostegård, 2013). Despite advancements in scientific research and medical technology, an effective method for the complete resolution of atherosclerotic plaques and the restoration of a normal vascular environment remains elusive. (Libby et al., 2016). Herein, we postulate the imperative to undertake rigorous, multifaceted investigations into the underlying mechanisms governing the disappearance of atherosclerotic plaques. The elucidation of these intricate physiological processes shall pave the way for discovering novel, more efficacious anti-atherosclerotic pharmacological agents. Consequently, this endeavor promises to enhance the curative outcomes for affected individuals, ultimately mitigating the substantial socioeconomic burden imposed by cardiovascular afflictions.

## 3 Mechanism of AS plaque regression

Overall, the progression of atherosclerosis involves a complex interplay of various cell types and inflammatory factors, leading to the formation of atherosclerotic plaques. The intricate pathogenesis of AS necessitates an equally complex regression mechanism. Moreover, the human body operates as a complex organic network, indicating that the regression of AS plaques is not solely influenced by individual factors or pathways, but rather by the interplay of various factors working in concert to either promote or reverse the progression of the disease.

### 3.1 Lipid reduction

Dyslipidemia is a primary causative factor in the formation of AS lesions and constitutes a significant characteristic of AS. Consequently, regulating the concentration of plasma lipoprotein to return to normal levels is the primary method of AS treatment.

Lowering the level of AS-causing lipoprotein, especially serum low-density lipoprotein cholesterol (LDL-C), can effectively reduce the overall AS plaque load and change plaque composition (Kataoka et al., 2017). In clinical practice, potent lipid-lowering statin drugs are widely used to diminish lipid levels in AS patients, serving as a cornerstone in the treatment regimen. These drugs reduce the plasma cholesterol levels of patients by inhibiting 3-hydroxy-3-methylglutaryl coenzyme A reductase (HMG-CoA), a key enzyme in the cholesterol biosynthetic pathway, thereby exerting their anti-AS effect (Almeida and Budoff, 2019; Sarraju and Nissen, 2024). Since 2016, an increasing number of non-statin drugs with lipid-lowering and anti-AS effects have been discovered and are frequently utilized in clinical practice for anti-AS treatment, either in combination with statins or as standalone therapies (Masana et al., 2023; Nakano et al., 2023; Sucato et al., 2024), For instance, proprotein convertase subtilisin/kexin type 9 (PCSK9) inhibitors can diminish its binding to low-density lipoprotein receptors (LDLR) on the surface of hepatocytes by targeting PCSK9, thereby increasing the quantity of LDLR, facilitating the clearance of LDL-C in plasma, lowering LDL-C levels, and exerting a protective role against AS (Nicholls et al., 2022; Biccirè et al., 2023). Research has substantiated that the lipid-lowering efficacy of combined treatment with lipid-lowering drugs is significantly superior to that of statin monotherapy, and the impact on plaque regression is also more pronounced (Sucato et al., 2024).

Reducing the plasma lipid cholesterol level in patients with AS is a critical precursor to the effective treatment of AS and the regression of AS plaques. Lipid-lowering therapy has been shown to improve vascular endothelial function and increase plaque stability (Krone and Müller-Wieland, 1999; Tousoulis et al., 2002), making it a crucial strategy for reducing the risk factors associated with AS. Lipid-lowering agents, particularly statins and PCSK9 inhibitors, continue to play a pivotal role in therapeutic regimens due to their dual capacity for cholesterol reduction and their additional benefits, which include anti-inflammatory effects and the stabilization of atherosclerotic plaques. (Dimitriadis et al., 2024; Ueki et al., 2024). However, the onset of AS is driven by abnormally elevated blood lipid levels and endothelial dysfunction. Therefore, while reducing plasma lipoprotein concentrations, it is imperative to promote the regeneration and functional repair of vascular endothelial tissue in areas that have been damaged.

### 3.2 Endothelial repair

Normal arteries possess a well-developed three-layer structure consisting of the intima, the media, and the adventitia arranged from the innermost to the outermost layer. The vascular endothelium, the innermost component of the blood vessel, is composed of a single layer of endothelial cells that, together with the basement membrane, form the vascular intima (Pugsley and Tabrizchi, 2000; Krüger-Genge et al., 2019). The vascular endothelium serves not only as a mechanical barrier between blood and surrounding tissues but also as a crucial endocrine organ. By sensing hemodynamic changes and blood-borne signals, it regulates vasomotor function through the release of vasoactive substances, thereby maintaining vascular homeostasis (Krüger-Genge et al., 2019).

The surface of vascular endothelial cells is comprised of a layer of glycoproteins that play an important role in essential physiological processes, such as endothelial cell signal transduction, material transport, and immune response (Pugsley and Tabrizchi, 2000). The functional integrity of endothelial cells is vital for maintaining vascular homeostasis. Endothelial dysfunction arises when there is an imbalance between vasodilator and vasoconstrictor substances secreted by endothelial cells, coupled with chronic mechanical shear stress resulting from conditions such as dyslipidemia, hypertension, or diabetes (Nafisa et al., 2018; Le Master et al., 2020). This dysfunction leads to increased permeability of vascular endothelial cells and abnormal expression of inflammatory factors (Tribe and Poston, 1996). In the early stage of AS, substantial lipid accumulation beneath the vascular endothelium results in endothelial cell damage and subsequent dysfunction. Therefore, promoting the functional repair of damaged vascular endothelium is a crucial step in the treatment of anti-AS.

In general, when the body’s intrinsic repair mechanisms are functioning effectively, endothelial damage can be repaired, and vascular integrity can be restored. These mechanisms typically entail cell replication and the replacement of non-functional endothelial cells (Yang et al., 2018). Current research suggests that endothelial cell repair involves two main mechanisms. On the one hand, existing mature endothelial cells may undergo mitosis, but their proliferation capacity is limited as most of them are terminally differentiated cells. The second mechanism involved in endothelial repair is via circulating endothelial progenitor cells (EPCs) (Woywodt et al., 2002). EPCs represent a circulating subset of vascular endothelial cells that are crucial for maintaining the stability of vascular endothelial structure and function. These cells are capable of differentiating and producing new endothelial cells, which helps in repairing damaged endothelial tissue. EPCs play a significant role in preserving the homeostasis of the vascular environment (Woywodt et al., 2002; Komici et al., 2023). These immature progenitor cells possess robust proliferation and differentiation capacities. In instances of endothelial tissue damage, they migrate to the affected site requiring repair, facilitating the proliferation and regeneration of endothelial cells within the damaged area or differentiate into mature endothelial cells via utilizing paracrine signaling pathways to support endothelial repair and preserve vascular homeostasis (Bai et al., 2010; Yao et al., 2013; Zhang et al., 2014).

Preserving endothelial barrier integrity is essential for upholding vascular homeostasis, regulating the fluid balance between circulating substances and surrounding tissues, and preventing the occurrence of vascular diseases. Repairing damaged endothelial cells to ameliorate endothelial dysfunction, along with lipid-lowering therapy, is critical for blocking factors that contribute to AS. However, while these strategies are essential, they are undoubtedly far from sufficient for the stability and regression of plaques. A comprehensive treatment for the regression of AS plaques should also focus on the processes involved in plaques regression and modifications in their composition.

### 3.3 VSMCs phenotypic regulation

VSMCs are important cellular components in the pathological progression of AS. It can undergo transdifferentiates into various types of cells under pathological conditions, such as macrophage-like cells, foam cells, osteoblast-like cells, mesenchymal stem cell-like cells, and myofibroblast-like cells. This transdifferentiation significantly influences the development and process of AS (Qi et al., 2019; Wang et al., 2023).

The phenotypic transformation of VSMCs *in vivo* is often affected by integrating molecular and environmental factors. Transcription factors such as myocardin (MYOCD), serum response factor (SRF), Krüppel-like factor 4 (KLF4), and octamer-binding transcription factor (OCT4) are notably significant in governing the phenotypic transition of VSMCs. These transcription factors are recognized as key regulators of the phenotypic transformation of VSMCs (Ackers-Johnson et al., 2015; Kansakar et al., 2021). MYOCD acts as a cofactor of SRF, binding to the CArG-box element in the promoters of contraction-related genes, thereby promoting the expression of VSMC contraction genes (Xia et al., 2017). Furthermore, the upregulation of MYOCD expression has protective effects on AS lesions by inhibiting plaque growth. Xia et al. (Xia et al., 2021) indicated that MYOCD can modulate the expression of ATP-binding cassette transporter A1 (ABCA1) in human aortic vascular smooth muscle cells (HAVSMCs) via an SRF-dependent mechanism to impact cholesterol transport processes. Knocking out MYOCD in ApoE^−/−^ mice resulted in reduced cholesterol efflux and increased intracellular cholesterol content due to the downregulation of ABCA1 expression. This dysregulation contributed to the exacerbation of atherosclerosis and facilitated plaque formation.

Several transcription factors, including KLF4, NF-κB, specific protein-1 (SP-1), and transcription factor 21 (TCF21), exert inhibitory control over the expression of genes associated with smooth muscle contraction. Their mechanisms involve disrupting the interaction between SRF and CArG-box, thereby initiating the dedifferentiation process of contractile smooth muscle cells (SMCs). Conversely, the maintenance of the contractile phenotype is positively governed by transforming growth factor β (TGFβ), miR143, and miR145. Notably, these molecules can cooperatively contribute to the partial downregulation of KLF4, thereby counteracting its influence in promoting smooth muscle cells dedifferentiation. This underscores their critical function in modulating the phenotype of smooth muscle cells (Grootaert and Bennett, 2021; Kansakar et al., 2021).

KLF4, a pivotal transcription factor involved in the phenotypic transformation of VSMCs, comprises three distinct domains: transcriptional repression, transcriptional activation, and DNA binding domains (Yang et al., 2021; Yap et al., 2021). The DNA-binding domain features a highly conserved cysteine 2/histidine two zinc finger structure, which facilitates specific recognition and binding to GC-rich DNA sequences within the promoter regions of target genes. This domain is essential for regulating critical cellular functions such as proliferation, differentiation, and apoptosis. Additionally, the presence of a transcriptional repression domain in conjunction with an N-terminal transcription activation domain enables KLF4 to switch between activating and inhibiting the transcription of target genes (Yoshida and Hayashi, 2014). VSMCs-specific knockout of KLF4 has been shown to significantly reduce plaque size and enhance plaque stability by impeding the transformation of smooth muscle cells into macrophage-like cells. This finding highlights the critical role of KLF4 in maintaining VSMC phenotype and its potential implications in atherosclerosis progression and plaque vulnerability (Shankman et al., 2015; Alencar et al., 2020).

**FIGURE 2:**
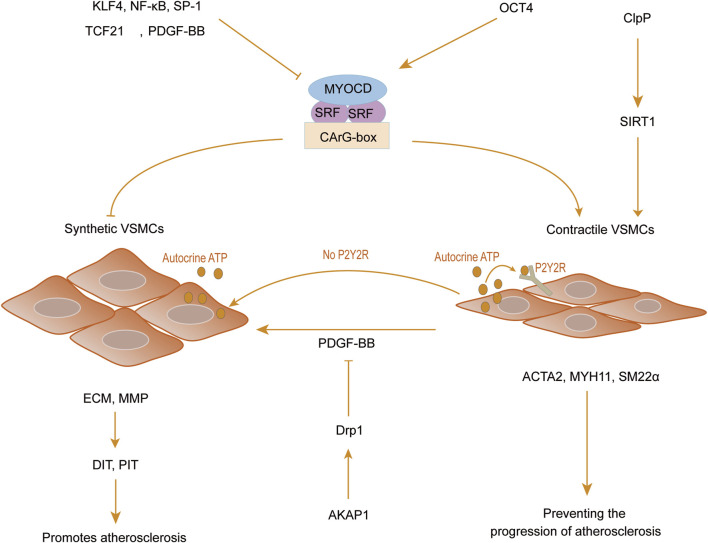
VSMCs phenotypic regulation. Myocardin (MYOCD) acts as a cofactor for serum response factor (SRF), binding to the CArG-box element located in the promoter regions of contraction-related genes to facilitate the upregulation of vascular smooth muscle cell contraction genes. Transcription factors like Krüppel-like factor 4 (KLF4), NF-κB, specific protein-1 (SP-1), transcription factor 21 (TCF21), platelet-derived growth factor BB (PDGF-BB), and the absence of P2Y2 receptor can drive the transformation of SMCs into a synthetic phenotype, consequently fostering excessive proliferation and migration of SMCs, exhibiting a pro-atherosclerotic effect. In contrast, octamer-binding transcription factor (OCT4), caseinolytic protease proteolytic protein (ClpP), and A-kinase anchoring protein 1 (AKAP1) exert a protective effect against atherosclerosis by preserving the expression of the contractile phenotype in smooth muscle cells.

OCT4, another pivotal pluripotency factor, plays a significant role in regulating phenotypic transitions and exerts an antagonistic effect on KLF4 (Cherepanova et al., 2016). The study conducted by Shin et al. (Shin et al., 2023) used an Oct4-IRES-GFP reporter mouse model and a tamoxifen-inducible Myh11-CreERT2 Oct4 knockout mouse model and found that the targeted knockout of OCT4 in VSMCs was observed to reduce the expression levels of key contractile markers including ACTA2, MYH11, and tagln. This reduction was associated with enhanced migration and excessive proliferation of VSMCs. The results indicate that OCT4 can be promptly upregulated in response to vascular injury, effectively modulating the expression of ACTA2 to impede phenotypic transitions and mitigate the excessive proliferation of VSMCs, ultimately preventing aberrant thickening of the vascular wall. In addition to the aforementioned regulatory factors, recent studies have revealed that VSMCs can autonomously release low levels of adenosine triphosphate (ATP) to modulate vasoconstriction. Chen et al. (Chen et al., 2024) observed that autocrine ATP release hinders the transition of VSMCs to a synthetic phenotype. This was evidenced by experiments inhibiting ATP release in human aortic smooth muscle cells, which showcased that the activation of P2Y2 receptors (P2Y2R) through basal ATP release is crucial for preserving the contractile phenotype of VSMCs. Furthermore, the role of mitochondria as central players in energy metabolism and their involvement in the phenotypic transformation of SMCs has become increasingly recognized. The research conducted by Sun et al. (Sun et al., 2024) discovered that the mitochondrial PKA anchoring protein A-kinase anchoring protein 1 (AKAP1) can inhibit PDGF-BB-mediated phenotypic transformation, proliferation, and migration of VSMCs by promoting Dynamin-related protein 1 (Drp1) phosphorylation (Ser637) via rat carotid artery balloon injury model and HAVSMCs treated with PDGF-BB *in vivo* and *in vitro*. In parallel, Paredes et al. (Paredes et al., 2024) employed primary human aortic smooth muscle cells (HASMCs) and a mouse model of elastase-induced aneurysm formation to demonstrate that caseinolytic protease proteolytic protein (ClpP), a mitochondrial protease, can govern the phenotype of VSMCs by elevating the NAD^+^/NADH ratio and activating the NAD^+^-dependent deacetylase Sirtuin1 (SIRT1). This action aids in upholding the contractile phenotype and differentiation state while diminishing the expression of VSMC dedifferentiation markers. These findings underscore the critical role of energy metabolism and mitochondrial homeostasis in modulating the VSMC phenotype. Consequently, fine-tuning mitochondrial homeostasis and energy metabolism may offer a promising preventive strategy against atherosclerosis by regulating the phenotypic transition of VSMCs. (Figure 2).

VSMCs demonstrate remarkable plasticity. Vessel injury induces a phenotypic transformation from differentiated to dedifferentiated VSMCs, which involves reduced expression of contractile proteins and increased production of extracellular matrix and inflammatory cytokines (Liang et al., 2024). This pathological phenotype switching plays a major role in the development of atherosclerosis and plaques (Li et al., 2013). Preserving the contractile phenotype of VSMCs and impeding their transition to a synthetic phenotype is crucial in halting vascular intimal thickening in the progression of AS. This approach can mitigate the formation and build-up of foam cells, thereby averting the advancement of AS.

### 3.4 Reverse cholesterol transport

The deposition of lipoproteins beneath the endothelium plays a crucial role in the development of atherosclerosis, with foam cells abundant in cholesterol esters emerging as a distinctive feature of atherosclerotic lesions. Reverse cholesterol transport (RCT), the mechanism of ferrying surplus cholesterol from peripheral tissue cells (including foam cells within atherosclerotic plaques) to the liver for breakdown and elimination from the body (Ohashi et al., 2005), has garnered significant interest as a potential strategy for halting or potentially reversing atherosclerotic lesions.

Lipid-bound cholesterol internalized by macrophages undergoes hydrolysis within lysosomes, yielding free cholesterol that is subsequently shuttled to and integrated into the plasma membrane. Surplus membrane cholesterol and a fraction of free cholesterol derived from low-density lipoprotein are directed to the endoplasmic reticulum, where they undergo re-esterification by acyl-CoA: cholesterol acyltransferase-1 (ACAT1) to form cholesterol ester (CE), which is then stored in the cytoplasm as lipid droplets to avert cytotoxicity stemming from excess free cholesterol. However, excessive accumulation of CE promotes foam cell formation in macrophages, subsequently accelerating the progression of AS (Ghosh, 2011). Hence, fostering the reverse transport of cholesterol within macrophages is imperative in thwarting foam cell formation and the progression of atherosclerotic lesions.

However, only free cholesterol can undergo efflux to facilitate reverse cholesterol transport (RCT). Therefore, promoting the hydrolysis of CE is not only the initial step in the occurrence of RCT but also the rate-limiting step in this process (Ouimet and Marcel, 2012; Ghosh, 2012; Sakai et al., 2014; Hafiane et al., 2019; Ouimet et al., 2019). The hydrolysis of intracellular CE can be classified into neutral CE hydrolysis and acidic CE hydrolysis due to the distinct pH levels in the reaction milieu (Jeong et al., 2017). Key enzymes participating in neutral CE hydrolysis include hormone-sensitive lipase (Lipe), carboxylesterase 3 (Ces3), and neutral CE hydrolase 1 (NCEH1), with NCEH1 assuming a primary function (Sakai et al., 2014). Furthermore, studies indicate that lysosomal acid lipase (LAL) also possesses the capability to hydrolyze CE. In the context of lipid accumulation, autophagy facilitates the transport of cytoplasmic lipid droplets to lysosomes within macrophages. Within the lysosomal lumen, LAL catalyzes the breakdown of lipid droplet CE, yielding free cholesterol for elimination (Jeong et al., 2017; Ouimet et al., 2019; Korbelius et al., 2023).

**FIGURE 3:**
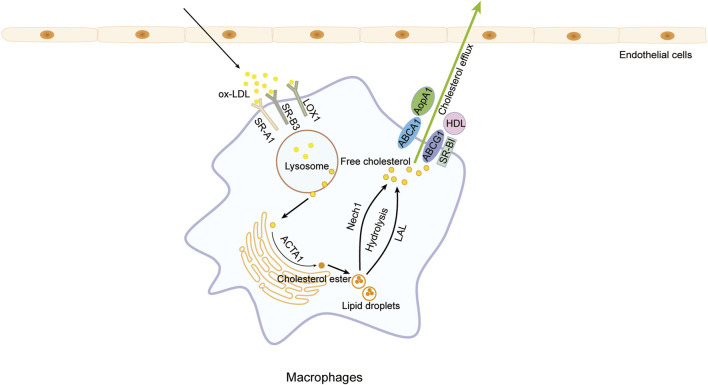
Cholesterol efflux. Macrophages phagocytize lipids through scavenger receptor A1 (SR-A1), SR-B3 (CD36), and lectin-like oxidized low-density lipoprotein (LDL) receptor-1 (LOX1), resulting in excessive lipids that drive macrophages to transform into foam cells., Cholesterol efflux can be enhanced to diminish intracellular cholesterol levels in macrophages by promoting the hydrolysis of cholesterol esters into free cholesterol and upregulating the expression of high-density lipoproteins (HDL) and apolipoprotein A1 (ApoA1), consequently reducing foam cell formation and safeguarding against atherosclerosis.

Free cholesterol is extricated from macrophages within the endothelium of the blood vessel wall via various mechanisms, including the action of ATP-binding cassette (ABC) transporters A1 and G1 (ABCA1/G1), passive diffusion, scavenger receptor B1 (SR-B1), and caveolins, among others. Subsequently, the liberated free cholesterol is ensnared by high-density lipoproteins (HDL) and apolipoprotein A1 (ApoA1) for conveyance to the liver for excretion (Ouimet and Marcel, 2012; Favari et al., 2015; Ouimet et al., 2019). ABCA1/G1 serves as pivotal receptors in the initial phase of RCT within atherosclerotic plaques (Yvan-Charvet et al., 2007), actively facilitating the transfer of cellular cholesterol from lipid-laden macrophages to extracellular receptors. Augmenting the expression of HDL or ApoA1 has been shown to mitigate the necrotic build-up of foam cells (Kluck et al., 2023). (Figure 3)

Macrophages uptake excessive lipids, leading to their transformation into foam cells. The buildup of lipid-laden foam cells beneath the vascular endothelium stands as a primary factor driving the expansion and progression of AS plaques. Facilitating the reverse transport of cholesterol within foam cells and diminishing the lipid burden of macrophages are intricately linked to fostering plaque regression. A recent study suggested that targeting Epsins, a family of endocytic adaptors, in lesional macrophages may offer therapeutic benefits for advanced atherosclerosis by reducing CD36-mediated lipid uptake and increasing ABCG1-mediated cholesterol efflux (Cui et al., 2023). Additionally, injection of recombinant HDL or apoA-I mimetic peptides accelerates cholesterol efflux from peripheral tissues, improves vascular endothelial state, and leads to regression of atherosclerotic plaque (Chiesa and Sirtori, 2003; Poteryaeva and Usynin, 2022). These studies indicate that enhancing reverse cholesterol transport within plaques may hold significant potential for facilitating plaque regression.

### 3.5 Removal and reduction of necrotic core

In the context of apoptotic foam cells or necrotic cores, timely removal of apoptotic cell remnants holds particular significance. Concurrently with enhancing cholesterol efflux from foam cells, the organism’s clearance mechanisms, and phagocytic capacities should be actively engaged to forestall the accumulation of apoptotic cells and the further enlargement of necrotic cores.

The necrotic core formation in AS lesions is attributed to the accumulation of cellular debris resulting from apoptotic necrosis of lipid- and cholesterol-laden foam cells. Under physiological conditions, apoptotic cell fragments are typically engulfed by phagocytic macrophages via efferocytosis, thus averting the release of apoptotic cellular contents and accumulation of debris (Westman et al., 2020), However, during the progression of AS, macrophages adopt a foam cell phenotype due to excessive lipid accumulation, which impairs efferocytic and phagocytic functions. This reduction in clearance capacity for apoptotic cells culminates in the accumulation of apoptotic cells and subsequent secondary necrosis. Consequently, the accumulation of cellular debris and release of cellular contents may exacerbate the progression, enlargement, and potential rupture of AS plaques (Kasikara et al., 2021; Li et al., 2022; Puylaert et al., 2022). Thus, enhancing the phagocytic clearance capacity of macrophages represents a crucial strategy for preventing the development of AS lesions and promoting plaque regression.

Macrophages migrate toward the vascular endothelium and phagocytose lipid particles, ultimately transforming into foam cells. The accumulation of these foam cells serves as a prerequisite for the development and progression of atherosclerotic plaques. Notably, macrophages exhibit substantial heterogeneity in their behavior and function during the development of atherosclerotic lesions. As the microenvironment within the plaque undergoes dynamic changes, the phenotypic profile of macrophages adapts accordingly. Distinct macrophage subtypes occupy dominant positions at various stages of atherosclerosis, reflecting their specific roles and contributions (Tabas and Bornfeldt, 2016). These diverse macrophage phenotypes play crucial roles in either promoting or inhibiting disease progression, thereby significantly impacting the overall development and evolution of atherosclerotic lesions.

#### 3.5.1 Polarization of macrophages

Monocyte-derived macrophages play a pivotal role in the pathogenesis of atherosclerosis, encompassing initiation, progression, and the eventual development of unstable plaques (Yang et al., 2024). These cells exhibit remarkable plasticity, capable of manifesting pro-inflammatory or anti-inflammatory properties, as well as reparative functions, contingent upon the specific microenvironment (Tabas and Bornfeldt, 2016). Extensive research has validated that macrophages can differentiate into distinct phenotypes, including the M1 phenotype with pro-inflammatory properties and the M2 phenotype with anti-inflammatory characteristics (Shapouri-Moghaddam et al., 2018). With advancements in macrophage research and technological progress, an increasingly diverse array of macrophage phenotypes has been discovered and characterized. Under various stimulatory conditions, macrophages can adopt phenotypes, such as Mox, which responds to oxidized phospholipids, as well as phenotypes sensitive to hemoglobin, encompassing hemoglobin-stimulated M(Hb), G-haptoglobin-induced HA-mac, heme-stimulated Mhem subsets, and M4 macrophages induced by CXC chemokine ligand 4. These diverse macrophage phenotypes contribute significantly to the complex pathogenesis of atherosclerosis (Barrett, 2020; Eligini et al., 2023).

M1 macrophages are polarized by microbial components such as lipopolysaccharide (LPS), granulocyte-macrophage colony-stimulating factor (GM-CSF), and interferon-gamma (INF-γ). They exhibit high expression of markers like CD80 and CD86 (Farahi et al., 2021), and drive inflammation by producing elevated levels of pro-inflammatory cytokines such as IL-1β, IL-6, IL-12, and TNF-α. Conversely, M2 macrophages are polarized by IL-4 and IL-13 and can be categorized into subtypes M2a, M2b, M2c, and M2d based on different stimuli. The activation of STAT6 through IL-4 receptor α by IL-4 or IL-13 induces macrophage polarization toward the M2 subset, while IL-10 triggers STAT3 via the IL-10 receptor to facilitate polarization of the M2 subset (Li et al., 2021; Li et al., 2022; Gianopoulos and Daskalopoulou, 2024). Unlike M1 macrophages, M2 macrophages exhibit high expression levels of markers such as CD206, arginase 1 (Arg1), and CD163. (Ira Tabas and Karin E. Bornfeldt, 2016; Barrett, 2020). They help dampen inflammation and facilitate tissue repair by producing anti-inflammatory cytokines like IL-10 and transforming growth factor-beta (TGF-β) (Shapouri-Moghaddam et al., 2018; Chen et al., 2020; Kadomoto et al., 2021).

**FIGURE 4:**
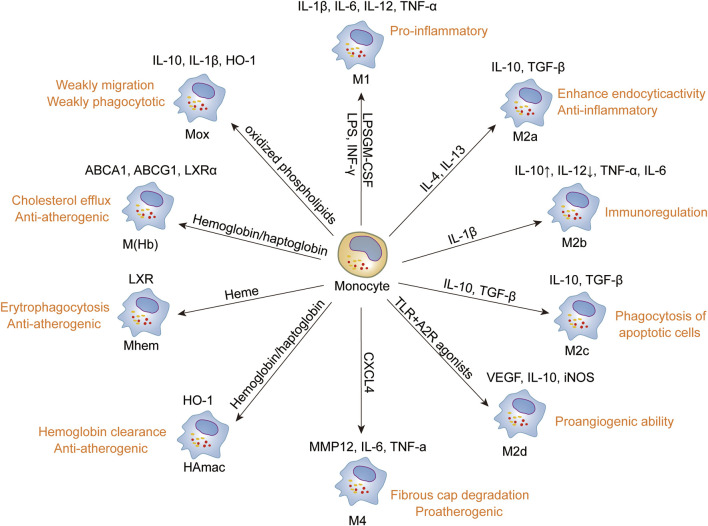
Phenotype and function of macrophages. Monocytes differentiate into macrophages upon infiltration into the plaque, displaying a spectrum of polarization states ranging from pro-inflammatory to anti-inflammatory phenotypes based on various stimuli, encompassing M1, M4, Mox, M(hem), M(Hb), M2a, M2b, M2c, and M2d. Each macrophage subtype is characterized by distinct stimuli, phenotypic markers, and functional attributes, reflecting their diverse roles in AS lesions and tissue homeostasis. Promoting the transformation of macrophages into anti-inflammatory or phagocytic phenotypes may be advantageous for the treatment of atherosclerosis and the induction of plaque regression.

Jablonski et al. (Jablonski et al., 2015) conducted an extensive analysis of the transcriptional profiles of mouse macrophages, identifying distinct sets of common and specific genes associated with M1 and M2 phenotypes. Notable findings included M1-specific genes such as CD38, Gpr18, and Fpr2, alongside M2-specific genes like c-Myc and Egr2. The CD38^+^Egr2^-^ phenotype is characteristic of M1 macrophages, whereas M2 macrophages typically exhibit CD38^-^Egr2^+^ traits. These observations suggest that the relative expression levels of CD38 and Egr2 can serve as distinguishing markers between M1 and M2 macrophage subsets. (Figure 4).

In the intricate milieu of AS lesions, macrophages exhibit a spectrum of phenotypes rather than a singular state, with multiple phenotypes coexisting at the lesion site. The predominant subsets of macrophages vary across different phases of disease progression. Within progressive plaques, M1 macrophages prevail, conversely, in stable plaques and the vascular adventitia where inflammation has waned, M2 macrophages are more prevalent (Blagov et al., 2023). Within the vascular intima, M1 macrophages undertake lipid engulfment, transitioning into foam cells, which in turn drives inflammation and facilitates further polarization of macrophages towards the M1 phenotype. As atherosclerotic lesions advance, the emergence of a necrotic core hastens inflammation and necrosis. M1 macrophages dominate in the lipid core proximal to the necrotic region, while M2 macrophages cluster in the neovascular region. Notably, M2 macrophages may migrate towards M1 macrophages to engulf them, thereby resolving inflammation and assuming an anti-AS role (Dri et al., 2023). Hence, fostering the polarization of macrophages towards the M2 phenotype characterized by anti-inflammatory and reparative properties, whether within the lesion site or circulation, holds profound implications and promising research avenues for treating atherosclerosis (AS) and facilitating plaque regression. By dampening inflammation within the plaque region and enhancing reparative processes in the necrotic area, pivotal advancements can be achieved in the therapeutic realm of AS management.

#### 3.5.2 Macrophage efferocytosis

The accumulation of cell debris, left unchecked after the apoptosis of foam cells, is paramount in driving the development and degradation of AS plaques. Cell death is an inevitable fate for all cells, and apoptosis, as a form of programmed cell death, is essential for sustaining homeostasis within living organisms. Vital in preserving the integrity of the cellular microenvironment is preventing the release of apoptotic cell contents that may harm neighboring tissues, as well as promptly eliminating the debris resulting from cell death. Here, the phagocytic and clearance capabilities of macrophages serve as indispensable functions in this protective process.

Macrophage efferocytosis in AS plaques represents a key determinant in the progression or regression of these plaques (Dolfi et al., 2021). Specifically, efferocytosis refers to the intricate process through which macrophages eliminate cells undergoing programmed cell death. This critical mechanism prevents secondary necrosis of dying cells (Doran et al., 2020; Schilperoort et al., 2023; Zhu et al., 2024), thereby averting the release of potentially deleterious cellular contents that could incite inflammatory responses. Consequently, macrophage efferocytosis is of significant importance in the repair and restoration of damaged tissues within the body. This multi-step process is intricately regulated and influenced by a diverse array of signaling molecules, ensuring both precision and efficiency in its execution.

**FIGURE 5:**
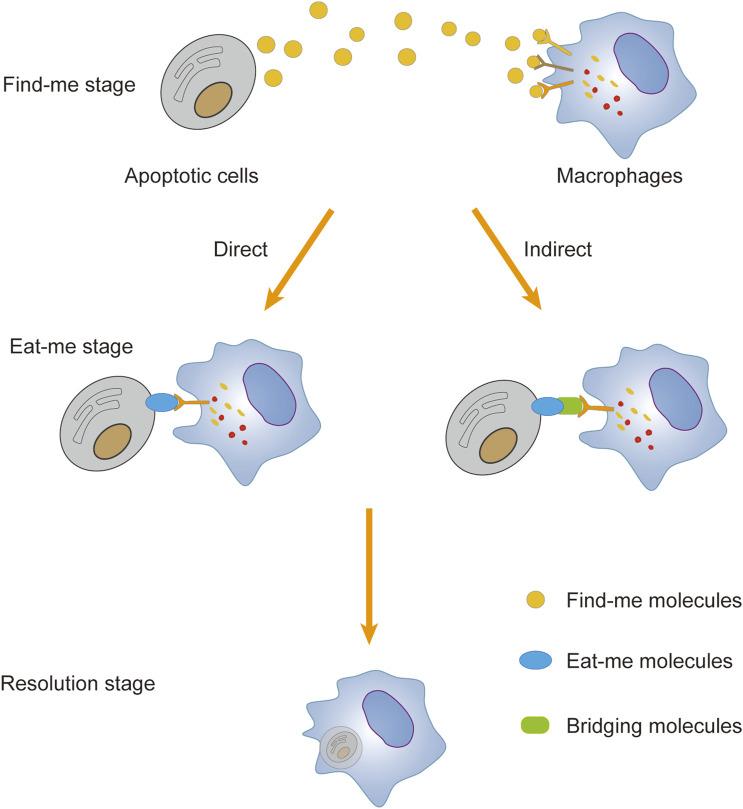
Process of macrophage efferocytosis. Apoptotic cells emit find-me signals to recruit macrophages, which subsequently identify eat-me signals on the apoptotic cell membrane and establish binding interactions directly or indirectly via bridging molecules. Subsequently, macrophages generate phagocytic cups to engulf apoptotic cells, leading to their internalization and subsequent breakdown to prevent the release of deleterious cellular contents. Apoptotic cells release find-me signals to recruit macrophages, which then detect eat-me signals on the apoptotic cell membrane and establish binding interactions either directly or indirectly through bridging molecules. Subsequently, macrophages form phagocytic cups to engulf apoptotic cells, facilitating their internalization and subsequent degradation, thereby preventing the release of harmful cellular contents.

In general, apoptotic cells emit a distinct series of “find me” signaling molecules, which include chemokines such as C-X3-C motif chemokine ligand 1 (CX3CL1), sphingosine-1-phosphate (S1P), lysophosphatidylcholine (LPC), and a minor fraction of adenosine triphosphate/guanosine triphosphate (ATP/UTP). These signaling molecules specifically bind to cognate receptors expressed on the surface of macrophages, effectively attracting and inducing the phagocytic congregation of macrophages near apoptotic cells. (Gonzalez and Trigatti, 2017; Gerlach et al., 2021; Razi et al., 2023). Macrophages possess the ability to recognize “eat me” signals, such as phosphatidylserine (PtdSer), calreticulin, oxidized low-density lipoprotein (ox-LDL), and intercellular adhesion molecule 3 (ICAM-3), which are presented on the membranes of apoptotic cells. This recognition is mediated by specific protein receptors, including stabilin 1, T cell immunoglobulin mucin receptor 1 (TIM1), and adhesion G protein-coupled receptor B1 (ADGRB1). The direct binding of these receptors to their cognate ligands activates macrophage phagocytosis in a “ligand-receptor” fashion (Shu et al., 2024). Additionally, macrophages can engage indirectly with bridging molecules and phagocytic receptors, thereby forming a “ligand-bridging molecule-receptor” complex that triggers the efferocytosis cascade. For instance, bridging molecules such as growth arrest-specific protein 6 (Gas6) and protein S link the externalized PtdSer on apoptotic cells to TAM receptor tyrosine kinases (TAMs), including tyrosine-protein kinase receptor 3 (TYRO3), tyrosine kinase receptor (AXL), and Mer tyrosine-protein kinase receptor (MerTK), thus initiating the clearance process (Chua et al., 2018; Myers et al., 2019). The bridging molecule milk fat globule-like epidermal growth factor 8 (MFG-E8) links PtdSer expressed on apoptotic cells to integrin M and αVβ5 (Chen K. et al., 2021). Furthermore, the low-density lipoprotein receptor-related protein (LRP) interacts with PtdSer through the bridging molecule β-2-glycoprotein 1, enhancing macrophage phagocytosis of apoptotic cells (Maiti et al., 2008). Upon binding of the “eat me” signal to phagocytic receptors, it triggers the activation of Rac1, a member of the Rho family of small guanosine triphosphatases (GTPases). This activation leads to the reorganization of the macrophage cytoskeleton, promoting the formation of a “phagocytic cup” structure around the apoptotic cells. Such structural rearrangement facilitates the encapsulation and subsequent phagocytosis of the apoptotic cells by macrophages (Kim et al., 2017; King and Kay, 2019). This process ensures the digestion and decomposition of apoptotic cells, thereby preventing the release and accumulation of apoptotic cell debris and the associated adverse reactions, such as inflammation. (Figure 5).

Moreover, thriving living cells shield themselves from phagocytic cell engulfment by emitting “do not eat me” signals. Among the prominent “do not eat me” signal agents extensively studied are CD47, programmed cell death ligand 1 (PD-L1), and CD24. These molecules interact with their respective receptors: signal-regulatory protein α (SIRPα), programmed cell death receptor 1 (PD-1), and sialic acid-binding immunoglobulin-like lectin 10 (Siglec10) (Barkal et al., 2019; Li et al., 2022; Karizak et al., 2023; Khalaji et al., 2023). CD47 is particularly noteworthy as a well-characterized “do not eat me” signaling molecule, as it is widely expressed on the membrane of nearly all healthy cells. Upon interaction with SIRPα, CD47 triggers the phosphorylation of SH2-containing protein tyrosine phosphatase-1 (SHP-1) and SHP-2. This event inhibits myosin accumulation at the site of macrophage synapse formation and restricts cytoskeletal reorganization in macrophages near the phagosome, ultimately thwarting their phagocytic activity (Adkar and Leeper, 2024; Shu et al., 2024). While typically suppressed during apoptosis, CD47 exhibits an atypical upregulation in the context of atherosclerotic plaques. This aberrant expression allows apoptotic foam cells to evade macrophage-mediated efferocytosis, contributing to the accumulation of cellular debris (Adkar and Leeper, 2024). The study conducted by Gerlach et al. (Gerlach et al., 2019) revealed that treatment with anti-CD47 blocking antibodies to LDLR^*−/−*^ mice with established atherosclerosis decreased necrotic cores, and limited the accumulation of plaque necroptotic cells.

Facilitating the transition to anti-inflammatory macrophage phenotypes and boosting macrophage endocytosis are key strategies for bolstering atherosclerotic plaque stability and facilitating plaque regression (Bao et al., 2024). Additionally, appropriate inhibition of “do not eat me” signals associated with apoptotic cell expression in plaques may effectively facilitate the removal of apoptotic cell remnants, thereby reducing both the accumulation and enlargement of atherosclerotic plaques. Given the pivotal role of macrophages in the entirety of atherosclerosis progression, modulating macrophage functions to elicit anti-atherosclerotic effects holds substantial research promise and significance.

## 4 Summary

AS arises from the interplay of diverse pathogenic elements, yielding complex symptoms that necessitate equally intricate and varied treatment strategies. While individual treatment modalities may provide partial relief from disease severity, they do not constitute a definitive cure. Therefore, integrating diverse approaches that are advantageous for managing AS lesions and implementing a comprehensive treatment framework across all facets of the disease may yield unexpected and beneficial outcomes.

Lowering blood lipid levels in individuals with AS stands as a pivotal factor in halting AS progression. In a study by Bartels et al. (Bartels et al., 2015), LDLR^−/−^ mice subjected to a high cholesterol diet for 16 weeks were treated with ApoB antisense oligonucleotides or mismatch control antisense oligonucleotides. Their findings in mice revealed that enhancing endothelial barrier function against plasma LDL particles or diminishing the influx of new LDL particles into the bloodstream could ameliorate morphological lesions preceding foam cell reduction. This approach significantly curtailed the inflammatory response within the lesion sites.

Endothelial dysfunction exacerbates LDL permeation and deposition beneath the intima. Hence, decreasing circulating lipid levels, alongside repairing endothelial tissue, restoring endothelial function, and preserving vascular homeostasis, is advantageous in impeding LDL accumulation and mitigating foam cell formation. Furthermore, considering the role of vascular smooth muscle cells (VSMCs) in the foam cell pool within atherosclerosis, a deeper exploration of the mechanisms governing VSMC foam cell generation will facilitate the development of novel therapeutic interventions aimed at curtailing cardiovascular disease (Pryma et al., 2019). Maintaining the contractile phenotype of vascular smooth muscle cells (VSMCs), inhibiting the expression and transition of macrophage-like cells with reduced phagocytic capacity, particularly toward a synthetic phenotype, as well as curtailing the proliferation and migration of VSMCs are beneficial strategies for impeding plaque development. Conversely, interventions aimed at promoting plaque regression are intricately linked to macrophage metabolism, inflammation resolution, and efferocytosis within established plaques (Tabas and Bornfeldt, 2020). Enhancing the cholesterol efflux capability of foam cells and fostering the reverse cholesterol transport pathway are advantageous for transitioning foam cells into regular macrophages, thereby reinstating macrophage functionality to diminish foam cell buildup. Concurrently, encouraging macrophage polarization toward the M2 phenotype characterized by anti-inflammatory attributes and boosting macrophage efferocytosis aid in mitigating the inflammatory reaction within the necrotic regions of plaques. This process facilitates the clearance of cellular debris and necrotic cores resulting from foam cell apoptosis, thereby impeding atherosclerosis progression and fostering plaque regression (Chistiakov et al., 2017b). It is imperative to recognize that the anti-atherosclerosis as mentioned above pathways are not isolated mechanisms, as they frequently interplay with one another. For instance, ABCA1 serves a dual role in facilitating cholesterol efflux and enhancing macrophage efferocytosis through the regulation of “find-me” signaling molecules such as LPC and the exposure, release, and expression of “eat-me” ligands like PtdSer. This pivotal function positions ABCA1 as a critical link between cholesterol efflux and apoptotic cell clearance (Chen W. et al., 2021).

Moreover, recent investigations have unveiled that smooth muscle cell-derived cells in both mouse and human atherosclerosis manifest several tumor cell-like traits, including genomic instability, evasion of senescence, heightened proliferation, resistance to apoptotic signals, invasiveness, and activation of extensive cancer-associated gene regulatory networks. Utilization of niraparib, an anti-cancer agent targeting DNA damage repair, has shown promise in mitigating atherosclerosis progression and prompting regression of advanced lesions in murine models (Pan et al., 2024). This breakthrough not only enhances comprehension of atherosclerosis pathogenesis and progression but also unveils promising avenues for the development of targeted therapeutic interventions against the disease.

Notably, atherosclerotic progression or regression in a specific vascular bed in patients with coronary artery disease (CAD) does not always align with changes observed in other vessels (Suzuki et al., 2011). Differences in hemodynamic forces and vessel wall composition might result in inhomogeneous atherosclerotic changes occurring among various vessels in response to atherogenic clinical and biochemical factors (Costopoulos et al., 2019).

To summarize, plaque regression constitutes a coordinated process involving the clearance of foam cells and extracellular cholesterol deposition, suppression of vascular smooth muscle cell migration, proliferation, and phenotypic alterations, and the substitution of pro-inflammatory macrophages with anti-inflammatory phagocytes. These phagocytes aid in necrotic substance removal and facilitate tissue healing. Moreover, the recent comparison of atherosclerotic plaques to tumors has illuminated potential pathways for plaque regression treatment. Approaching AS plaque regression akin to tumor regression may challenge the prevailing notion that AS plaques are incurable, providing new opportunities for comprehensive management strategies.
